# Supplementary figures and images for: Accelerated bacterial detection in blood culture by enhanced acoustic flow cytometry (AFC) following peptide nucleic acid fluorescence in situ hybridization (PNA-FISH)
Source: PLoS One. 2019 Feb 8;14(2):e0201332. doi: 10.1371/journal.pone.0201332 (PMC6368374; doi:10.1371/journal.pone.0201332)

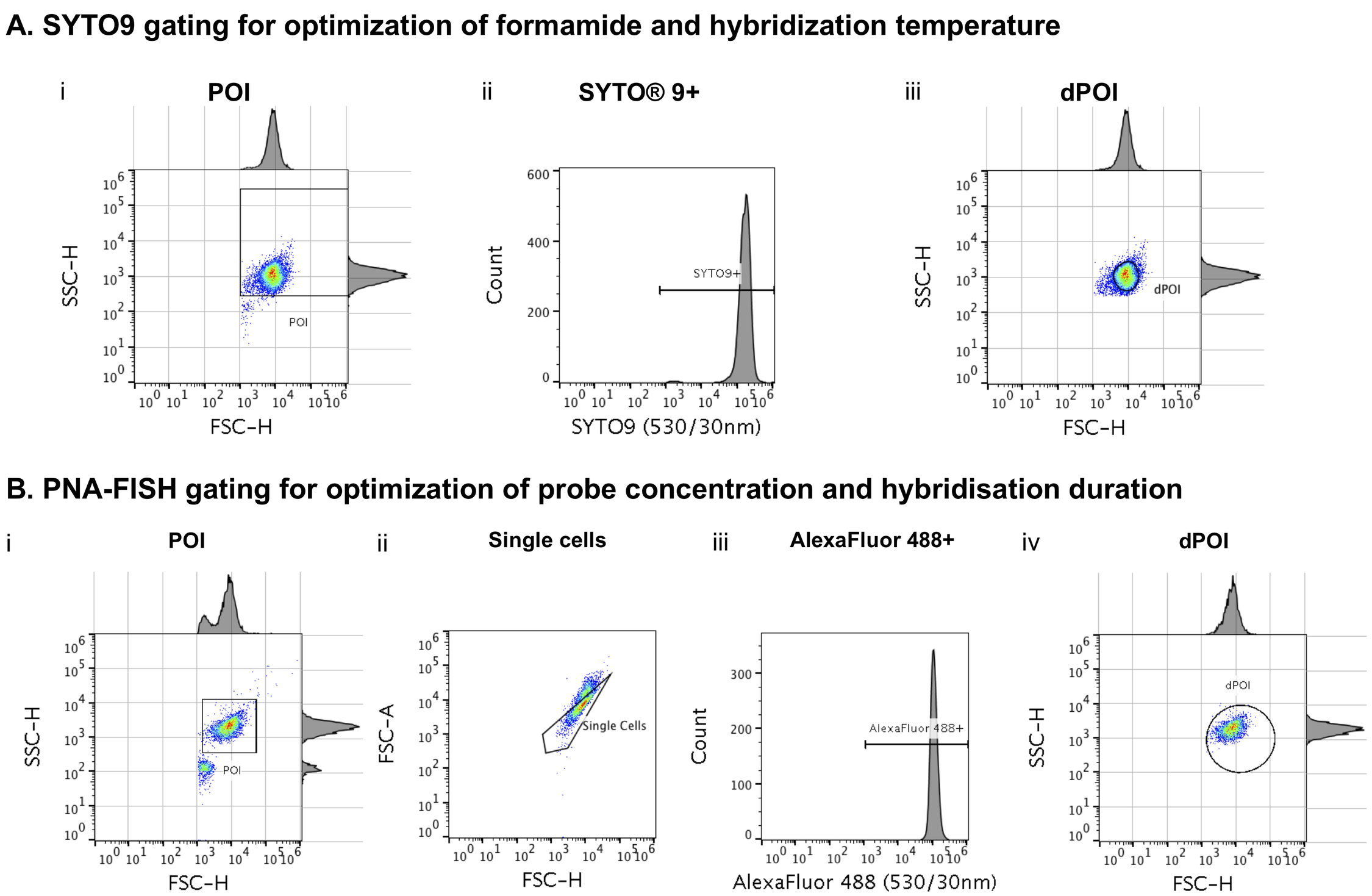

Supplement: S1 Fig — A: SYTO 9 was used in the formamide and hybridization temperature optimization experiments to investigate the overall bacterial population that contained both single and multiple events. POI was identified based on FSC-H/SSC-H plots, from which SYTO 9 positive population was identified on the histogram. A 10% contour was applied to the SYTO 9 positive population and labelled derived POI (dPOI). B: PNA-FISH gating on pure bacterial culture where POI was identified based on FSC-H/SSC-H plot followed by FSC-H/FSC-A to exclude doublet events. Probe specific events were identified from the singlet population as AlexaFluor 488+ on the histogram and this population was further gated as dPOI using FSC-H/SSC-H plot. (TIF) [file pone.0201332.s001.tif]

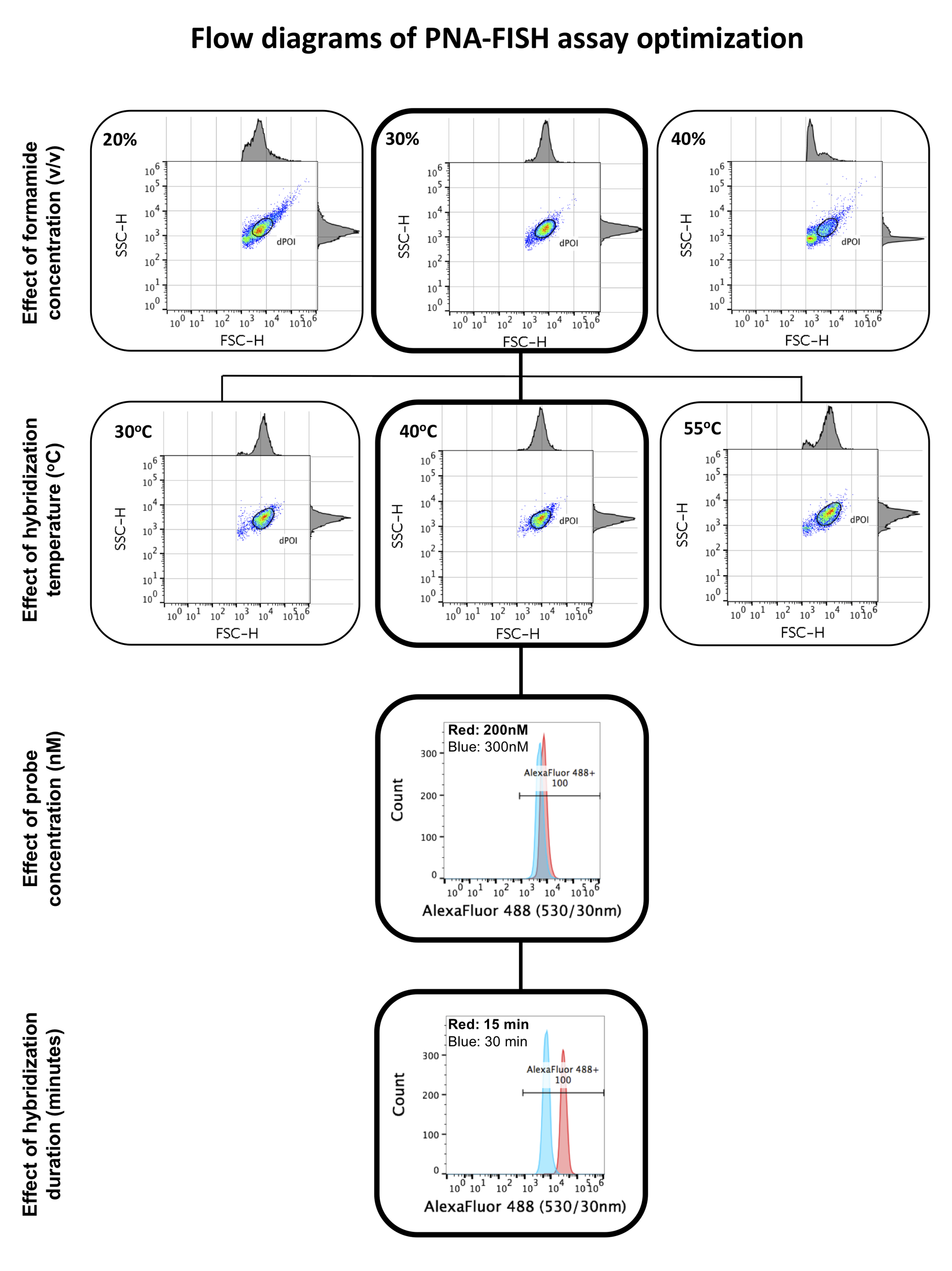

Supplement: S2 Fig — Four optimization experiments were set up sequentially to identify optimal conditions for bacterial detection by PNA-FISH-AFC as shown in Fig 3. Thick frames indicate the optimal conditions selected for subsequent experiments. Firstly, bacteria in pure culture were exposed to varying concentrations of formamide. The effect of increasing concentrations of formamide from 20 to 40% (v/v) on K. pneumoniae was monitored by SYTO 9 staining and presented as dPOI in the FSC-H/SSC-H plots (top row). The best cell preservation with tight bacterial population in the dPOI was observed in 30% formamide when compared to buffers containing 20 and 40% (v/v) formamide. In the second row, bacteria were hybridized in buffer containing 30% formamide (v/v) for 15 minutes at varied hybridization temperatures of 30°C, 40°C and 55°C followed by SYTO 9 staining. At temperatures of 30°C and 40°C, a better cell preservation was observed with minimal debris to the left of dPOI than at 55°C, where the debris subpopulation to the left of dPOI increased indicating likely degradation of bacterial cells at elevated temperature. In a third row, bacteria were hybridized at 40°C for 15 minutes in hybridization buffer containing 30% formamide (v/v) followed by two washes using wash buffer. Signal intensities of PNA-FISH probe concentrations of 200 nM and 300 nM were compared in a histogram where no significant difference between them was observed. Hence, 200 nM probe concentration was selected for the following experiment to test hybridization duration. In the last experiment (bottom row), two aliquots with the same concentration of bacteria were subjected to PNA-FISH assay for either 15 or 30 minutes under the conditions described above. The signal intensity was 1 log stronger after 15 minutes hybridization (red) than after 30 minutes (blue). Hence, the optimal hybridization condition for PNA-FISH-AFC was determined in the presence of 30% formamide (v/v) and 200 nM probe at 40°C for 15 minutes. (TIF) [file pone.0201332.s002.tif]

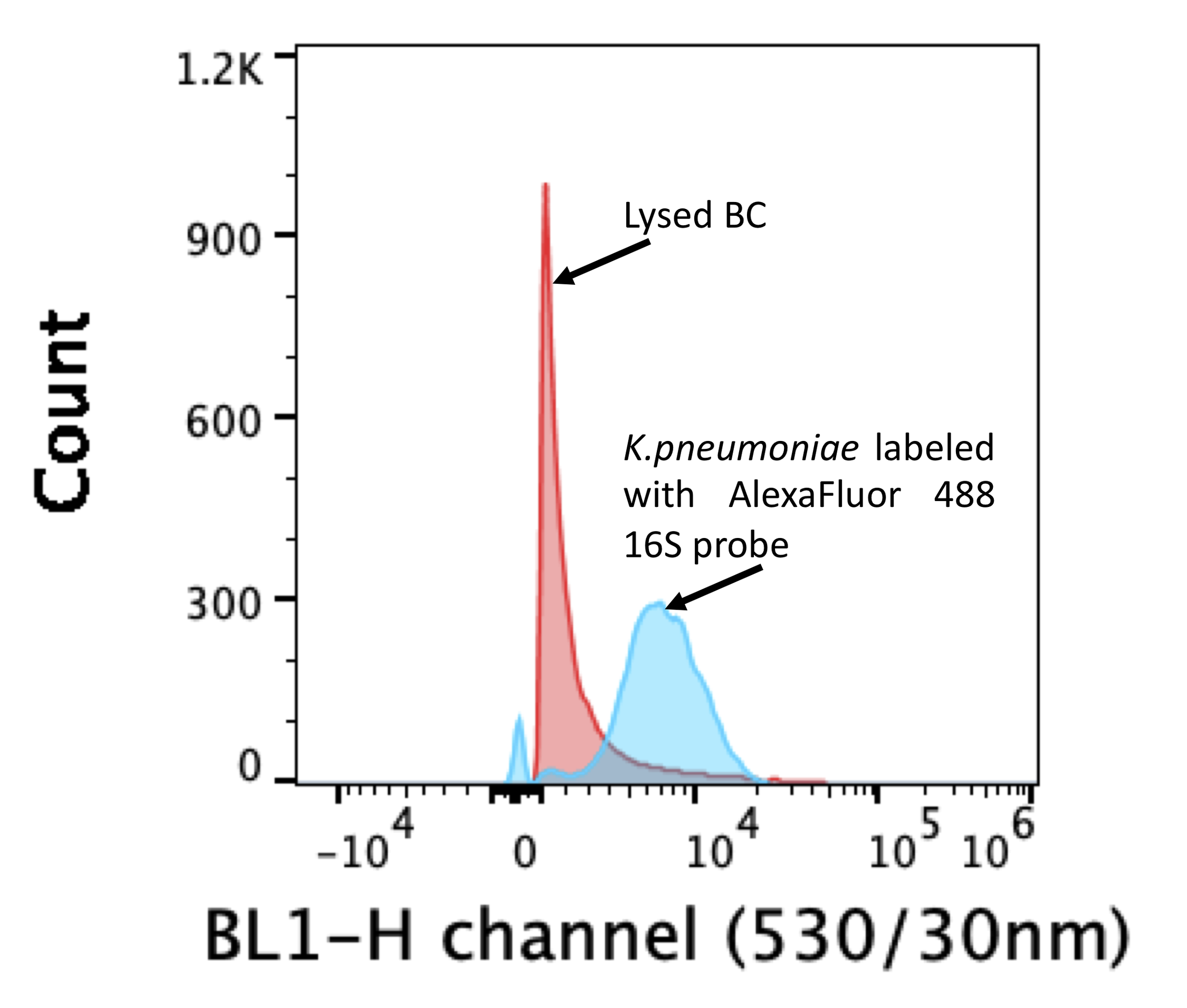

Supplement: S3 Fig — Unstained lysed BC (red) exhibited low levels of autofluorescence in the blue channel (BL1) where signals of AlexaFluor 488 labelled bacteria (blue) is collected. The false positive signal from the lysed BC within the overlapped region will skew the result of bacterial detection. Hence, a dual parametric gating method was developed (Fig 4) for better discriminations. (TIF) [file pone.0201332.s003.tif]

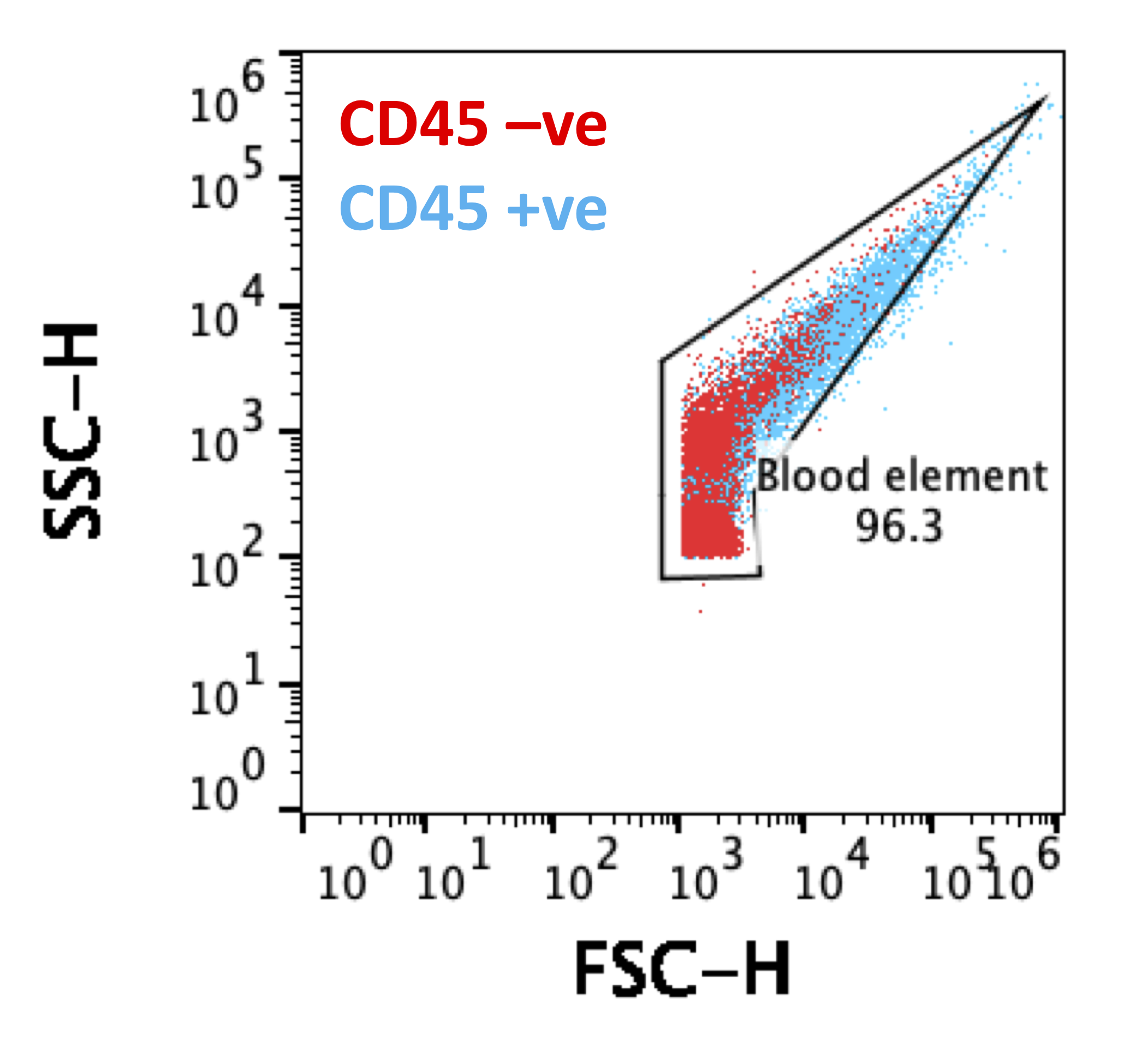

Supplement: S4 Fig — Overlay of CD45 positive and CD45 negative samples from lysed blood population in Fig 4i. The overlap in the same region indicates the population was of blood origin. (TIF) [file pone.0201332.s004.tif]

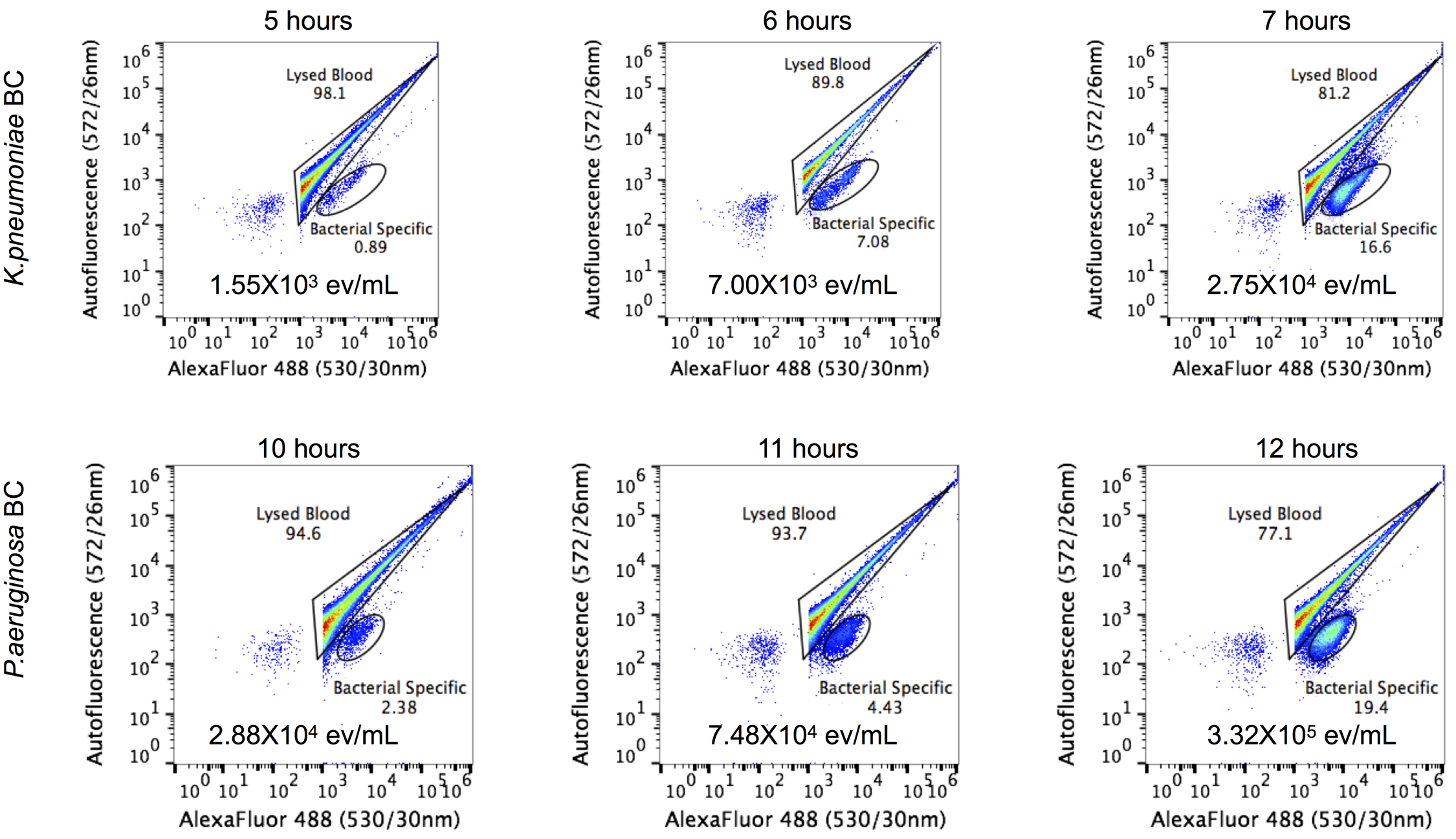

Supplement: S5 Fig — Similar to experiments with E. coli spiked BC (Fig 5), two other bacterial species, K. pneumoniae and P. aeruginosa were subjected to PNA-FISH-AFC following extended incubation in BC. The lowest bacterial concentration detected for both species was between 103−105 ev/mL as shown below the bacteria-specific population on the plots. While K. pneumoniae was first detected by PNA-FISH at 5 hours of incubation on the BacTEC FX system (top row), it took 10 hours to detect P. aeruginosa (bottom row). Additional 2 hours of incubation resulted in an increase of bacteria specific population for both organisms. (TIF) [file pone.0201332.s005.tif]

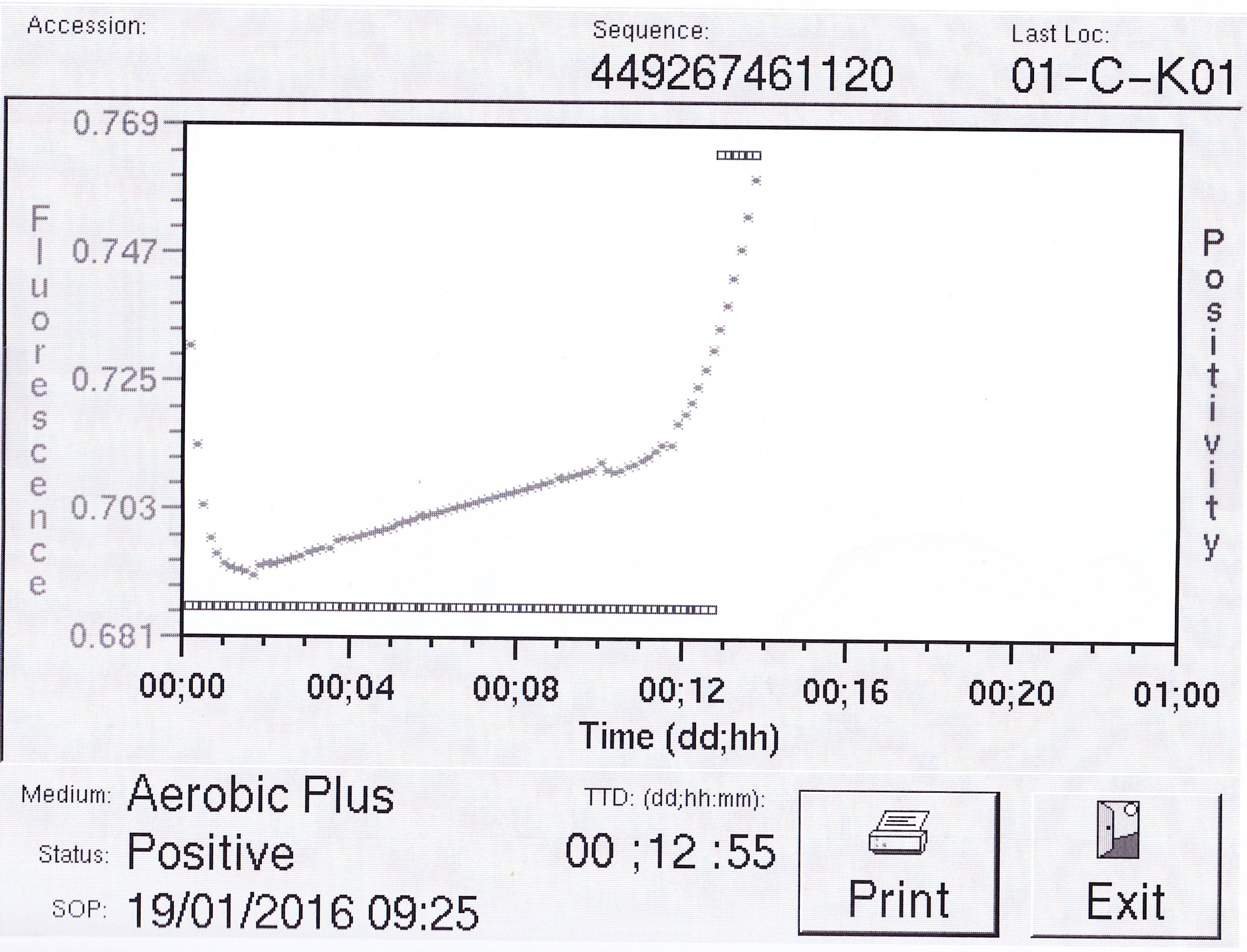

Supplement: S6 Fig — The growth curve generated on the BacTEC showing sampling for CO2 fluorescence every 5 minutes till threshold was reached. An increase of fluorescence was used to indicate bacterial respiration due to the release CO2. Time in hours was shown on the x-axis, while CO2 fluorescence was shown as arbitrary units on the y-axis. This particular BC culture was initially spiked with 10 CFU/mL E.coli and flagged positive after 12 hours and 55 minutes on the BacTEC FX system. (TIF) [file pone.0201332.s006.tif]
